# Periodontal Management of Gummy Smile Due to Altered Passive Eruption: A Case Report

**DOI:** 10.7759/cureus.29174

**Published:** 2022-09-14

**Authors:** Pavan Bajaj, Komal R Bhombe, Ranu R Oza

**Affiliations:** 1 Department of Periodontology and Implantology, Sharad Pawar Dental College and Hospital, Datta Meghe Institute of Medical Sciences, Wardha, IND; 2 Department of Periodontics and Implantology, Sharad Pawar Dental College and Hospital, Datta Meghe Institute of Medical Sciences, Wardha, IND

**Keywords:** altered passive eruption, esthetics, gummy smile, crown lengthening procedure, excessive gingival display

## Abstract

Today's population is expanding quickly, and there is a growing desire for aesthetics. Smiles and other friendly facial expressions communicate joy and assurance. They are the essential elements of nonverbal communication and play a significant part in establishing a person's first impression. The altered passive eruption, which results in the excessive gingival display (EGD) when the gingival edge is situated incisal to the cervical convexity of the crown, is one of the factors affecting aesthetics. It has an impact on the patient's appearance and grin. The management of EGD becomes crucial. The following case study covers the control of EGD with a crown lengthening operation.

## Introduction

The American Academy of Periodontology has identified gingival excess as a mucogingival malformation around teeth [1]. Patients may have serious aesthetic concerns with excessive gingival display (EGD), popularly known as a “gummy smile,” regardless of whether it is acquired or developing. Before beginning treatment, a thorough examination and diagnosis are necessary to determine the exact cause of EGD, including gingival enlargement, altered or delayed passive eruption, vertical maxillary excess, anterior dentoalveolar extrusion, short upper lip, hyperactive upper lip, and combinations thereof [2]. There are two stages to the eruption of a tooth: the active eruption stage, during which the tooth breaks through the gum tissue, and the passive eruption stage, during which the soft tissue covering the tooth's crown migrates apically [3]. The gingival border maintains a more coronal position, protecting more tooth enamel if a passive eruption does not advance. A neglected cause of the gummy smile is altered passive eruption. According to Goldman and Cohen, “altered passive eruption” is a disorder that only affects adults and causes the gingival border to be incisal to the cervical convexity of the crown [4]. Failure of tissue to reach cemento-enamel junction (CEJ) was defined as “delayed passive eruption” by Volchansky and Cleaton-Jones [5]. Due to the heterogeneity in diagnostic criteria, altered passive eruption's prevalence has increased from its previously reported 12.1% prevalence [5] to its current 35.8% prevalence [6].

A simple gingivectomy is indicated when it is judged that the osseous level is suitable; more than 1 mm separates the buccal bone crest from the cementoenamel junction, and an adequate height of connected gingiva will remain after surgery (type 1A) [7]. The breadth of the gingival keratinized band is often typical in type 2A. The crew of keratinized gingiva should be repositioned apically to a place at or near the cementoenamel junction to best treat individuals with type 2A [8]. Most patients with altered passive eruption and thicker buccal bone, necessitating osteoplasty, according to Zucchelli, respond best to an apically positioned flap [9]. Ostectomy is recommended when the results of the diagnostic procedures show osseous levels close to the cementoenamel junction level. This treatment is frequently linked to a flap that is positioned apically. Since the origin of an EGD determines how to treat it. This case report's objective is to summarize the source, diagnosis, and surgical treatment of a case with a gummy smile brought on by a changed passive eruption.

## Case presentation

**Figure 1:**
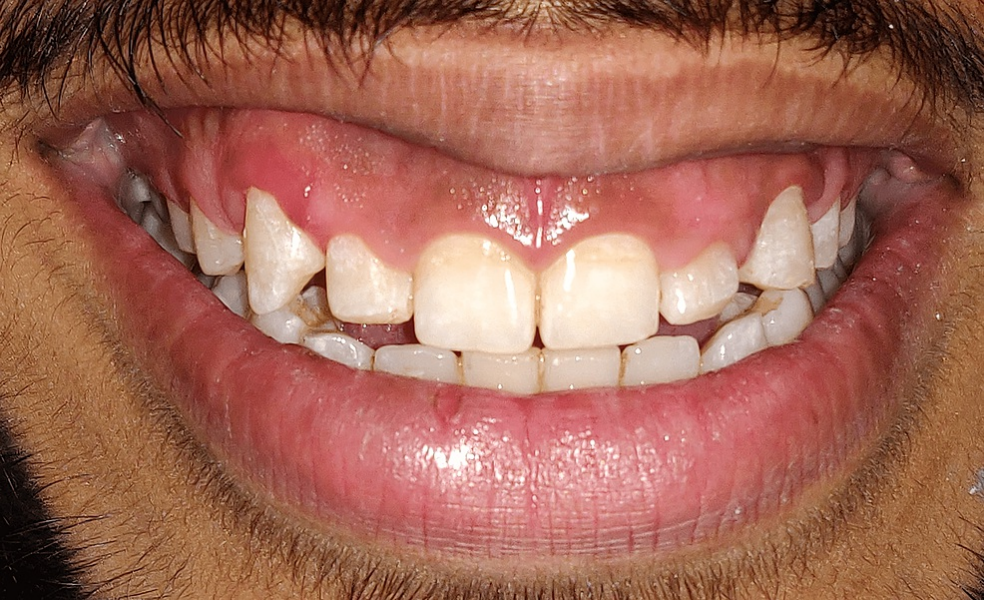
Pre-operative view depicting patient smile on day 1

**Figure 2:**
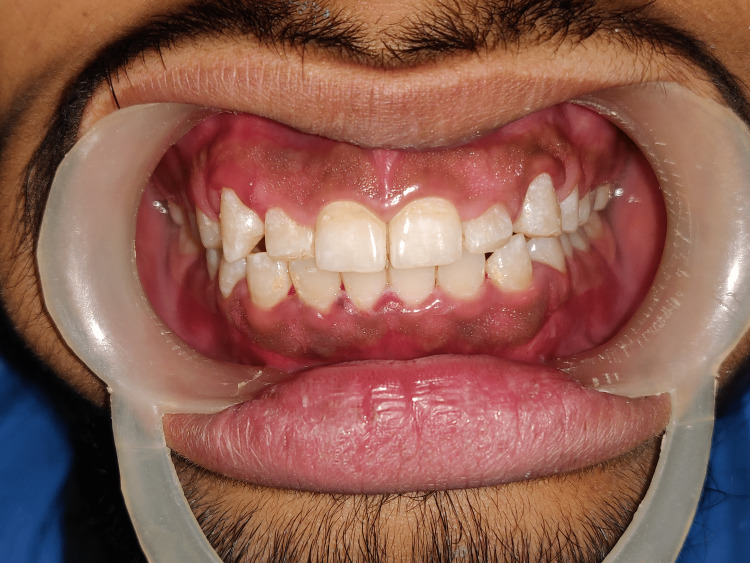
Intra-oral examination depicting the hard and soft tissues

A 27-year-old male patient visited the outpatient Department of Periodontics and Implantology, Sharad Pawar Dental College (DMIMS [DU]) with the chief complaint of a gummy smile and improper aesthetics. The patient provided a past orthodontic treatment history for closing gaps. Following an extraoral and intraoral examination, the patient was evaluated to make the proper diagnosis and develop a treatment plan. Following extraoral assessment, it was noticed that the patient had competent lips at rest, average lip length and mobility, and a standard facial height without any vertical maxillary excess, as indicated in Figures 1, 2.

**Figure 3:**
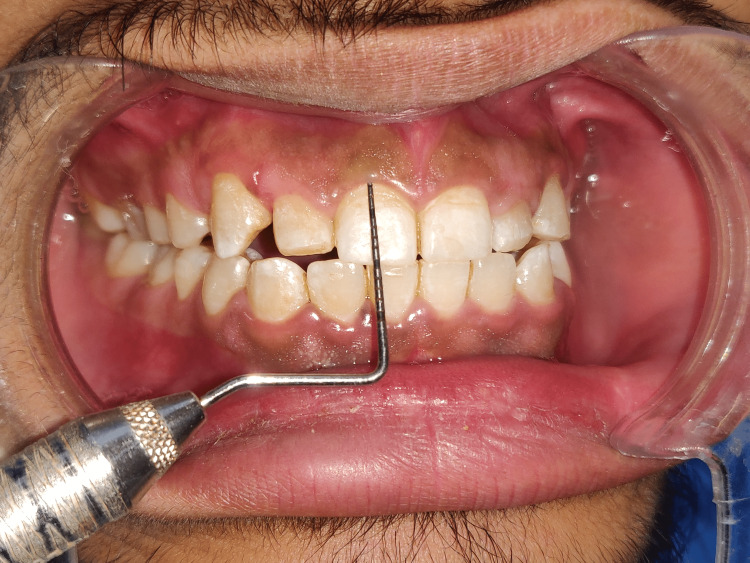
Clinical crown measurement of right central incisor as 8mm (11)

**Figure 4:**
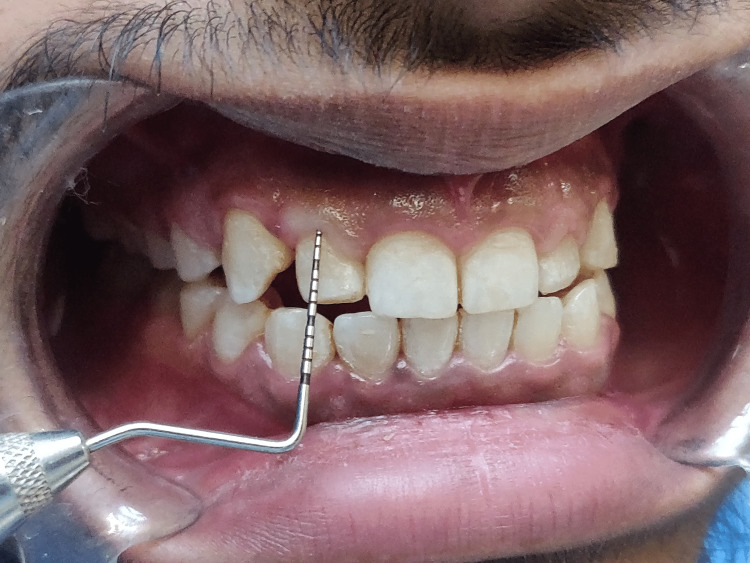
Clinical crown measurement of right lateral incisor as 6mm (12)

**Figure 5:**
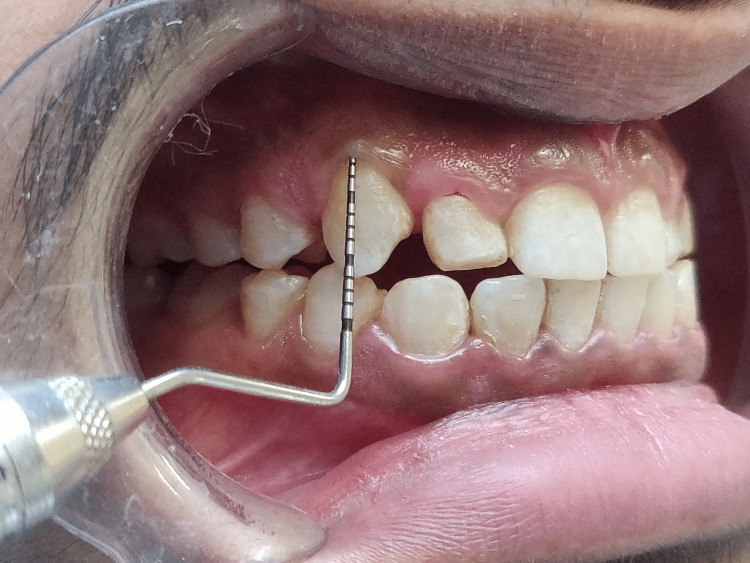
Clinical crown measurement of right canine as 7mm (13)

**Figure 6:**
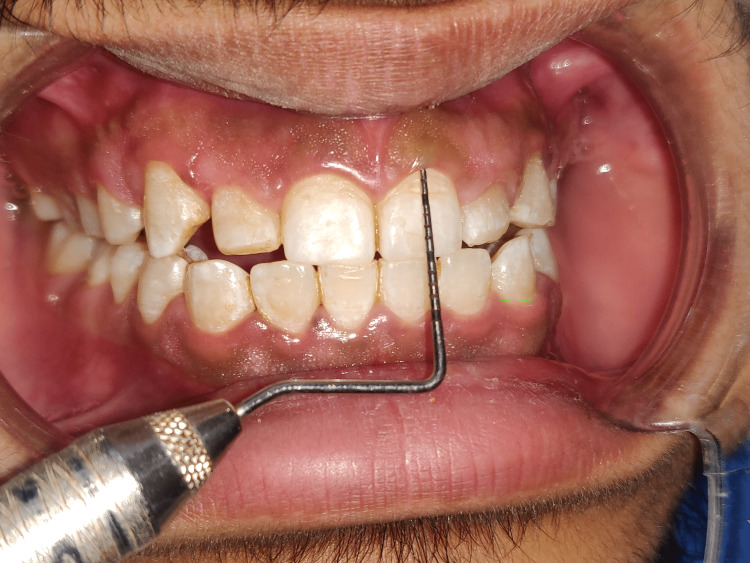
Clinical crown measurement of left central incisor as 7mm (21)

**Figure 7:**
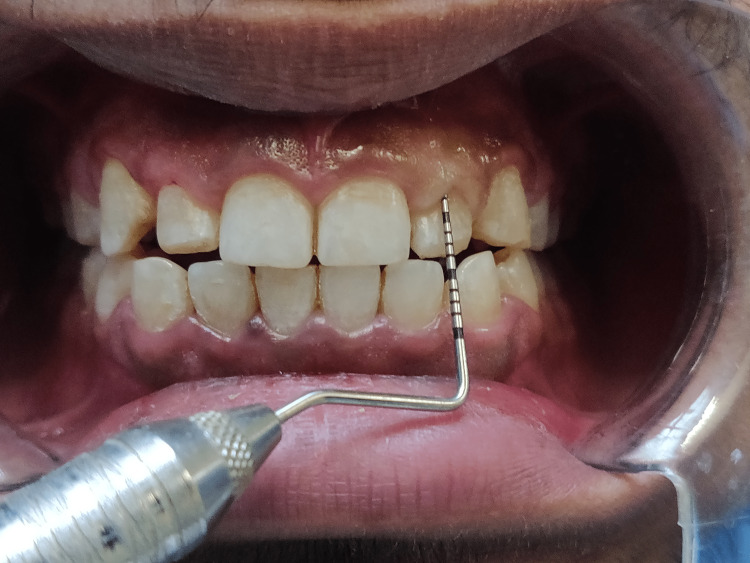
Clinical crown measurement of left lateral incisor as 5mm (22)

**Figure 8:**
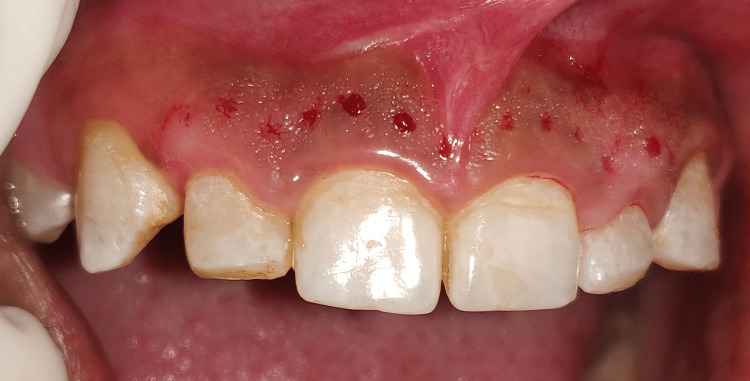
Marking depicting bleeding spots after pockets

Short clinical crowns measured on the mid-buccal aspect of the height from the gingival margin to the incisal edge were found during the clinical examination to have healthy gingiva and periodontium (right central incisor - 8mm, right lateral incisors - 6mm, right canine - 7mm, left central incisor - 7mm, left lateral incisor - 5mm, left canine - 7mm) as depicted in Figures 3-8. As a result, the diagnosis of gummy grins due to altered passive eruption (type I - subgroup B) [9] was made, and it was determined to treat choice with aesthetic crown lengthening surgery with osseous reduction.

Management

**Figure 9:**
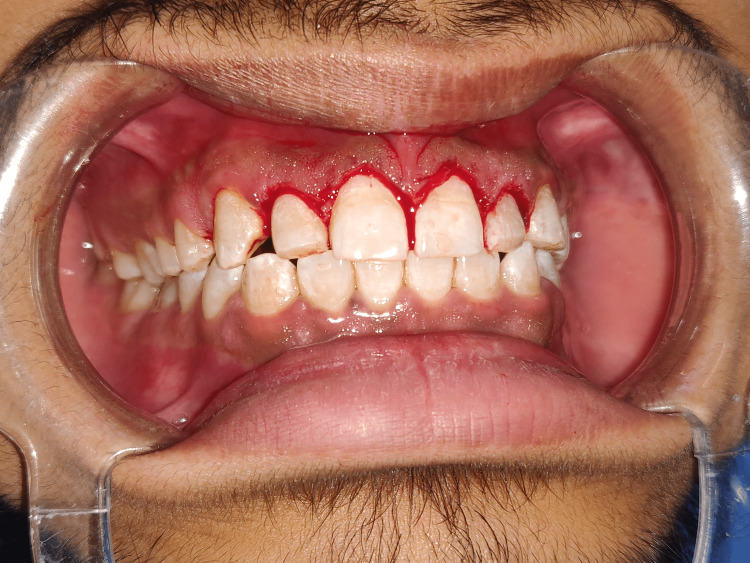
Internal bevel incision in place

**Figure 10:**
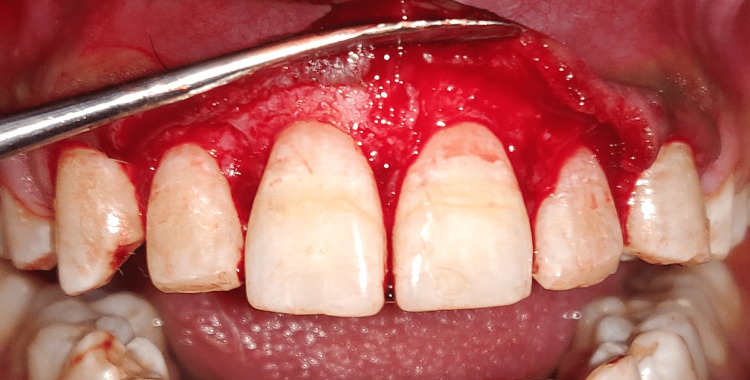
Full thickness mucoperiosteal flap raised

**Figure 11:**
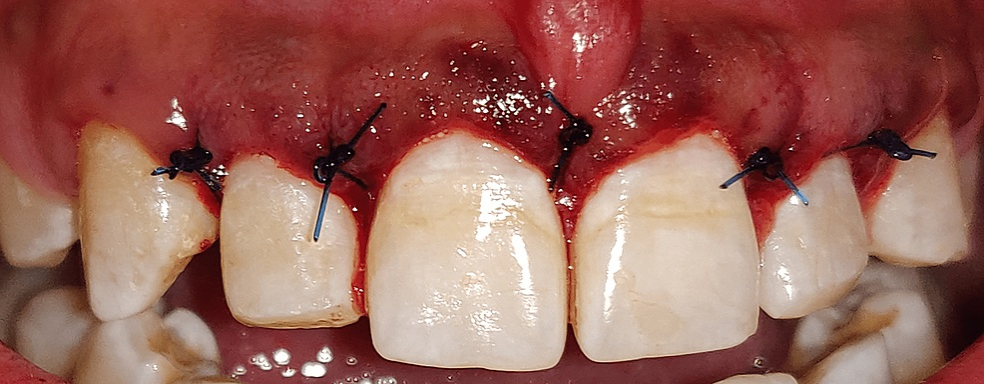
Placement of sutures after osseous reduction

Prophylaxis, oral hygiene teaching, and crown lengthening procedure (CLP) were all part of the treatment regimen. On the first visit, oral prophylaxis was done, followed by instructions on oral hygiene, and the patient was summoned back six weeks later for another evaluation and surgical treatment. Teeth 11-13 and 21-23 underwent aesthetic CLP. After administering a local anesthetic (2% Lidocaine, 1:100,000 epinephrine), bone sounding was carried out to confirm the site of the bony crest. A pocket marker was utilized to create bleeding spots, as shown in Figure 9. Then, a 15 C blade was used to make a scalloped submarginal internal bevel incision on the buccal side only, beginning from the mesial aspect of the right first premolar to the mesial aspect of the left first premolar, as shown in Figure 10. A full thickness mucoperiosteal flap was then reflected using a periosteal elevator after the gingival collar was produced, as shown in Figure 11.

 

**Figure 12:**
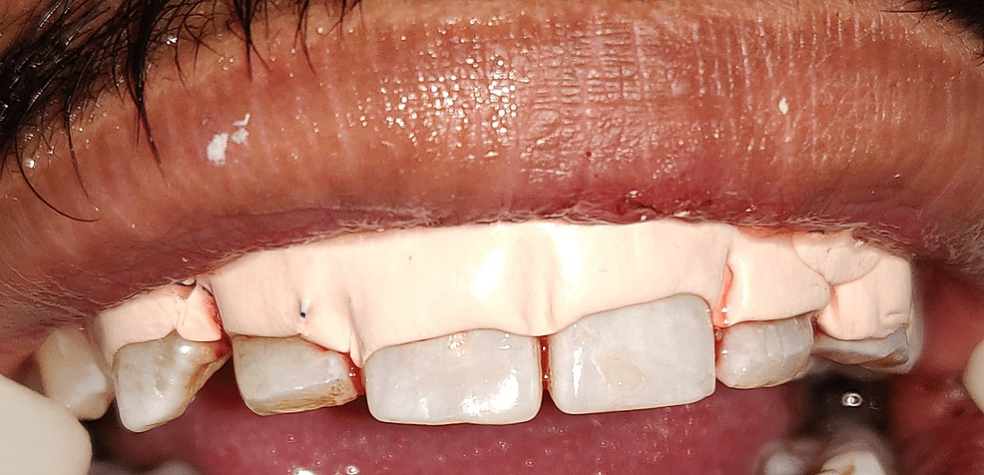
Placement of the periodontal pack

**Figure 13:**
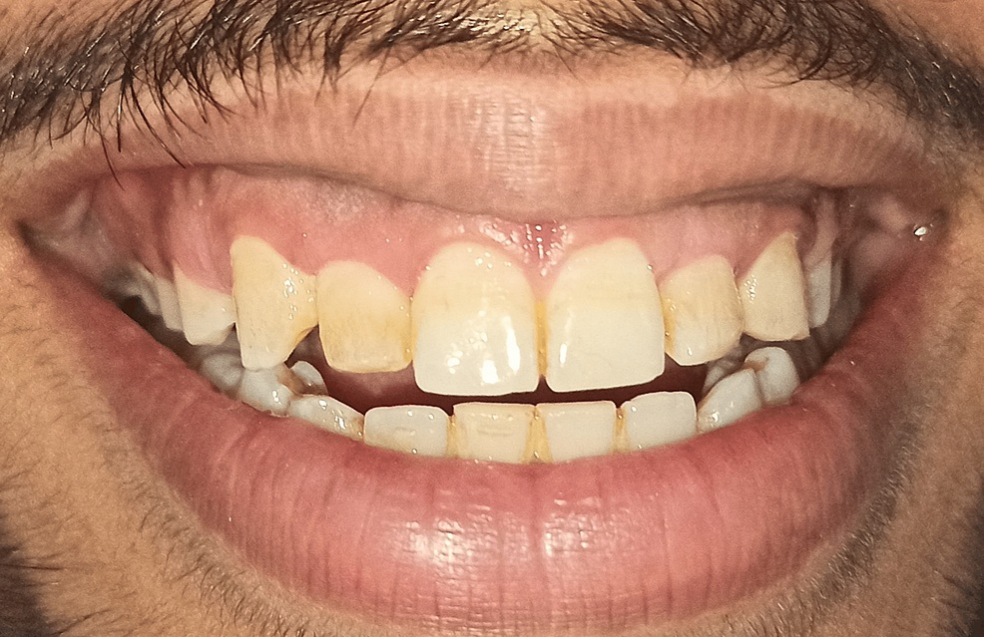
Postoperative clinical presentation after three months

To achieve the optimal maxillary anterior crown contours, osseous excision was used to move the bony crest 1-2 mm apical to the CEJ. The flap was sutured back together with non-resorbable sutures (Ethicon Vicryl Sutures USP 3-0, 1/2 Circle Cutting), and a periodontal pack (COE-PAKTM AUTOMIX) was applied to the surgical site. Figure 12 shows sutures and periodontal pack in place. Amoxicillin 500 mg TDS for five days, aceclofenac BD for three days, and 0.12% chlorhexidine rinse twice daily for two weeks were given to the postoperative patient recommendations included using an ice pack, eating soft foods, avoiding mechanical damage to the treated area, and moving your lips as little as possible. After two weeks, the stitches were removed. After surgery, the patient underwent careful follow-up for oral hygiene. Three months following surgery, the patient was reevaluated. Figure 13 shows the postoperative view after three months.

 

## Discussion

The current case study indicates how changed passive eruption-related gummy smiles can be successfully treated with the recommended medication. Gummy smile care varies from case to case and is determined by the condition's etiology and patient preferences. For the duration of the periodic follow-up, which lasted for three months, the patient had a noticeable improvement in the shapes of his anterior maxillary teeth and a decrease in the gingival display. A case study showed how to treat patients with gummy smiles by using the elevator muscles in the upper lip and nasal wing. This procedure reduced the elevation of the upper lip, correcting cosmetic changes to the smile and resulting in a decreased gingival display [10].

In many practices nowadays, aesthetic periodontal operations are as every day as reconstructive therapy was in the past due to the change in the clinical paradigm of periodontal treatment. In addition to complex tissue dimensions, incisor and gingival display are other elements that affect the aesthetics of a smile [11]. The positioning of the gingival zenith and the harmony of the gingival levels, in addition to the anterior maxillary teeth, are the essential components of a smile pleasing to the eye [12]. A perfect harmony of three factors - the white (teeth), pink (gum), and the lips - determines the beautiful grin. Many patients have experienced aesthetic shame due to excessive gingival shown when smiling, which impacts their psychosocial behavior [13].

## Conclusions

Predictable results in the treatment of EGD are obtained through periodontal plastic surgery and osseous surgery, respectively. Careful preoperative planning improves surgical outcomes, increases gingival margin stability after surgery, and satisfies patient aesthetic expectations. The effective management of EGD is influenced by a number of factors. Presurgical evaluation and diagnosis make up the first factor. Bone sounding can be used to locate the bone crest. Based on the bone margin to the CEJ or the eventual crown margin, the treatment options of gingivectomy or an apically positioned flap could be used alone or in conjunction with osseous surgery. It will require osseous surgery if it is less than 3 mm. The location of the initial incision to reveal the perfect tooth contour is the second crucial component. Third, the degree of bone reduction (ostectomy) following gingivectomy and flap reflection should match the desired amount of extra tooth length exposure.
